# The Genome of the Mitochondrion-Related Organelle in *Cepedea longa*, a Large Endosymbiotic Opalinid Inhabiting the Recta of Frogs

**DOI:** 10.3390/ijms232113472

**Published:** 2022-11-03

**Authors:** Weishan Zhao, Xialian Bu, Hong Zou, Wenxiang Li, Shangong Wu, Ming Li, Guitang Wang

**Affiliations:** 1 State Key Laboratory of Freshwater Ecology and Biotechnology, Institute of Hydrobiology, Chinese Academy of Sciences, No. 7 Donghu South Road, Wuhan 430072, China; 2 The Innovation Academy of Seed Design, Chinese Academy of Sciences, Wuhan 430072, China; 3 Protist 10,000 Genomics Project (P10K) Consortium, Institute of Hydrobiology, Chinese Academy of Sciences, Wuhan 430072, China; 4 University of Chinese Academy of Sciences, Beijing 100049, China

**Keywords:** *Cepedea longa*, mitochondrion-related organelle, organellar genome, stramenopile, phylogenetic analyses

## Abstract

Mitochondrion-related organelles (MROs) are loosely defined as degenerated mitochondria in anaerobic and microaerophilic lineages. Opalinids are commonly regarded as commensals in the guts of cold-blooded amphibians. It may represent an intermediate adaptation stage between the conventional aerobic mitochondria and derived anaerobic MROs. In the present study, we sequenced and analyzed the MRO genome of *Cepedea longa*. It has a linear MRO genome with large inverted repeat gene regions at both ends. Compared to *Blastocystis* and *Proteromonas lacertae*, the MRO genome of *C. longa* has a higher G + C content and repeat sequences near the central region. Although three Opalinata species have different morphological characteristics, phylogenetic analyses based on eight concatenated *nad* genes indicate that they are close relatives. The phylogenetic analysis showed that *C. longa* clustered with *P. lacertae* with strong support. The 18S rRNA gene-based phylogeny resolved the Opalinea clade as a sister clade to *Karotomorpha*, which then further grouped with *Proteromonas*. The paraphyly of Proteromonadea needs to be verified due to the lack of MRO genomes for key species, such as *Karotomorpha*, *Opalina* and *Protoopalina*. Besides, our dataset and analyses offered slight support for the paraphyly of Bigyra.

## 1. Introduction

Mitochondria are generally believed to be evolved from an endosymbiotic α-proteobacterium within an ancestral archaeal-derived host cell [1,2,3,4]. They exhibit diverse forms and are categorized into five classes based on the completeness of the electron transport chain (ETC) and energy metabolism: aerobic mitochondria, anaerobic mitochondria, hydrogen-producing mitochondria, hydrogenosomes and mitosomes [1,5,6]. The mitochondria belonging to the latter three classes are commonly called mitochondrion-related organelles (MROs), and they are often found in anaerobic or low-oxygen niches, such as gastrointestinal tracts, vaginas and anoxic sediments [7,8,9]. However, in recent years, many studies aiming to clarify MROs’ anaerobic metabolism and evolutionary relationships found that MROs might not be a strict classification with clear borders among classes but rather a spectrum of metabolic and functional phenotypes. For example, the flagellate *Monocercomonoides* sp. completely lost the mitochondrial genome [10,11,12,13,14]. Oxygen-restricted conditions are believed to be the major driving force for the transformation from aerobic mitochondria to MROs [8]. Mitochondria in classes 1–3 contain organelle genomes, but their size and contents are vastly reduced in contrast to the genomes of α-proteobacteria relatives [1].

The stramenopiles are an extraordinarily diverse group of eukaryotes, including photosynthetic lineages that range from diatoms to giant multicellular brown algae and nonphotosynthetic lineages that comprise free-living flagellates, parasites and organisms resembling fungi [15]. It also constitutes one of the most diverse clades of protists, which branches with Rhizaria and Alveolata within the SAR supergroup [15,16,17]. Several stramenopile taxa are parasites/commensals of Metazoa (e.g., *Blastocystis*, *Proteromonas*, *Cepedea*, *Opalina*, *Protoopalina*, *Aureococcus*, etc.) and plants (*Phytophthora*, etc.) [18]. For example, *Blastocystis* is a polymorphic and an unusual enteric protozoan parasite of humans and many other animals [19]. However, its pathogenicity is controversial since it is estimated that *Blastocystis* could be present in more than 1 billion humans, and it is commonly found in healthy individuals [20,21,22,23]. *Proteromonas* is an obligately anaerobic stramenopile that lives as a commensal in the intestine of urodelans, lizards and rodents [24,25]. Opalinids are commonly regarded as commensals in the guts of cold-blooded vertebrates, especially in the cloacae of amphibians [26,27]. Species belonging to the genus *Cepedea* are cylindrical and multinucleated, which distinguishes them from the other four genera of opalinids (*Protoopalina*, *Zelleriella*, *Opalina* and *Protozelleriella*) [26,28]. *Cepedea longa* was first discovered in the intestines of *Fejervarya limnocharis* (synonym: *Rana limnocharis*) and named by Bezzenberger [29]. The three lineages mentioned above exhibit different morphological forms. For example, there are three major forms (vacuolar, granular or ameboid) in *Blastocystis* without flagella, and the vacuolar form is considered the typical cell form [19,30]; the anterior part of *Proteromonas* possesses two flagella, one thicker and longer than the other [25,31]; and *Cepedea* is greatly elongated and cylindrical, as well as thickly flagellated on the cell surface [26]. However, species of the above-mentioned three opalinid lineages are phylogenetically relatively close (on the basis of 18S rRNA sequences) and form a monophyletic group within the Opalinata [27,31,32]. Mitochondria with tubular cristae were observed at the periphery of *C. longa* cells [26], which resemble the MROs in *Blastocystis* sp. and *P. lacerate*. The ancestor of Opalinidae and *Blastocystis* was most probably free-living since their closest relatives are all free-living [27,33]. In addition, *Blastocystis* is better adapted to anaerobic conditions than Opalinidae [34]. On this basis, opalinids may represent an intermediate adaptation stage between the conventional aerobic mitochondria and derived anaerobic MROs [34].

Both *Blastocystis* and *Proteromonas* can be grown in a man-made medium axenically [25,35,36,37], so it is relatively easy to gain research materials for investigating the genetics and biology of these species [25,38,39]. Although there is no in-vitro culturing method for *C. longa*, its cells are large and distinguishable in the recta of frogs (*F. limnocharis*), so it is possible to increase the number of cells via multiple sampling to conduct sequencing and analysis. Herein, we sequenced and analyzed the MRO genome of *C. longa* and conducted comparative mitogenomic and phylogenetic analyses with other organelle genomes within the Opalinata lineage.

## 2. Results

### 2.1. Cepedea longa and Its MRO Architecture

**Figure 1:**
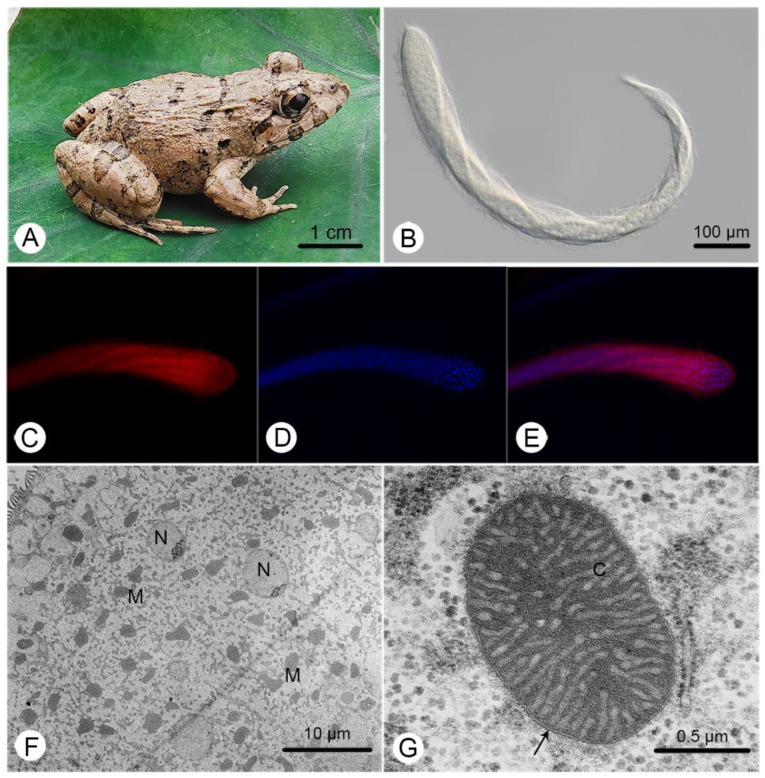
General characteristics of *Cepedea longa*, its host and its mitochondrion-related organelles. (**A**) The photo of the most common host of *C. longa*, *Fejervarya limnocharis*; (**B**) a microscopic image of *C. longa*; (**C**) a *C. longa* specimen stained with Mitotracker Red; (**D**) a specimen stained with DAPI; (**E**) a merger of (**C**,**D**); (**F**) a transmission electron microscope (TEM) image of *C. longa*, showing various MRO (M) shapes and ovoid nuclei (N); (**G**) a TEM image of the MRO of *C. longa*, showing the double-membrane (arrow) and cristae (C).

The body of *C. longa* is greatly elongated, slightly flattened, and thickly flagellated, with the cell surface twisted and coiled when moving (Figure 1B). A more detailed morphological description can be found in Li et al. [26]. Large amounts of MROs and nuclei were identified (Figure 1C,D), but the number of MROs was obviously larger than the number of nuclei (Figure 1E,F). They exhibited a range of shapes (Figure 1F) and double-membranes with highly convoluted cristae (Figure 1G).

**Figure 2:**
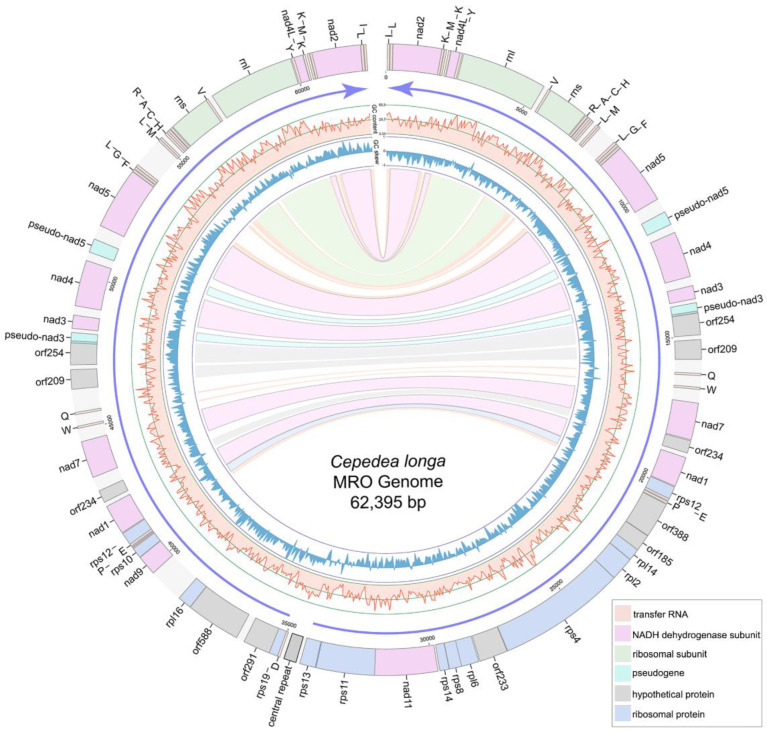
Gene map of the *Cepedea longa* mitochondrion-related organellar genome. Light blue blocks are ribosomal proteins; pink blocks are *nad* genes; gray blocks are unidentified ORFs; light orange blocks are tRNA genes; light green blocks are small/large subunit ribosomal RNA genes of MRO genome; grey block with a black box between the tRNA^Asp^ and *rps13* genes is the central repeat region of MRO genome. Purple arrows show the transcriptional direction. GC content and GC skew are shown as light red lines and blue lines, respectively. Repeat genes are indicated by corresponding colored links.

The MRO genome of *C. longa* is 62,395 bp-long with a linear structure. It contains two large inverted repeat regions with 11 protein-coding genes (PCGs), 20 tRNAs and 2 rRNAs each (Figure 2, Supplementary Table S1). The *C. longa* MRO genome contains 40 PCGs, 16 of which encode NADH dehydrogenase subunit genes; 13 PCGs encode ribosomal proteins; whereas 11 open reading frames (ORFs) remain unidentified (Figure 2, Supplementary Table S1). Only one ribosomal protein was identified within the two large repeat regions (*rps12*), whereas all other ribosomal proteins were found within the non-repeat (unique) region (Figure 2). The total length of 40 PCGs is 38,313 bp, with an average length of 958 bp and an average G + C content of 23.3%, varying from 17.24% of *orf291* to 31.91% of *nad7*. All PCGs used the ATG start codon, except for the *orf233* and *rpl16* genes. As regards stop codons, 27 were TAA, and 13 were TAG (Supplementary Table S1). There are conserved 5′ fragments with ATG start codon in both the pseudo-*nad5* gene (443 bp) and pseudo-*nad3* gene (333 bp). The pseudo-*nad5* has a degraded 3′ end, and the pseudo-*nad3* gene possesses an internal stop codon TAG (Supplementary Table S1).

A total of 41 tRNAs were detected in the MRO genome. Among these, 40 were located within the large repeat regions (Figure 2). Genes encoding tRNA^Thr^, tRNA^Ser^ and tRNA^Asn^ were absent from the MRO genome. Genes *rnl* and *rns* are 2764 and 1346 bp in size, with G + C content of 33.36% and 36.48%, respectively (Supplementary Table S1). A set of tRNA genes, tRNA^His^-tRNA^Cys^-tRNA^Ala^-tRNA^Arg^, was located ahead of *rns* (Figure 2), which was also detected in *P. lacertae*. There are 76 intergenic regions (from 1 bp to 1147 bp) interspersed within the MRO genome, with a total of 11,510 bp and an average of 151 bp (Table 1). Repeat regions add up to 1563 bp, accounting for about 2.5% of the MRO genome. The central repeats (several repeat units at the central position of the linear genome) of *C. longa* are 500 bp long in total, with five repeat units (Supplementary Table S3). Genes are arranged in the opposite transcriptional directions diverging from the central region, and GC skew values switch from negative to positive in the central region (Figure 2).

### 2.2. Comparison of MRO Genome Features in Opalinata Species

In accordance with *Proteromonas* and *Blastocystis*, no cytochrome b (*cob*), cytochrome oxidase subunits (*cox1*-*cox3*) and F_0_F_1_-ATPase subunits (*atp*) genes were found in the MRO genome of *C. longa* (Supplementary Table S2). The G + C content (25.7%) of the whole MRO genome is high in comparison to the other Opalinata species (18.9% to 22.7%), while the G + C content (25.4%) of concatenated intergenic regions (IGRs) is more than twice as high as others (8.4% to 12.0%) (Table 1). The Ka/Ks ratios (ω, non-synonymous substitutions/synonymous substitutions) for all PCGs of *C. longa* vs. *P. lacertae* ranged from 0.05 to 0.30 (Supplementary Figure S1). The functional constraints (negative selection) on *nad3*, *nad4L*, *rps4*, *rps10*, *rps13* and *rps19* genes were relaxed in comparison to other protein-coding genes. The selection pressure analysis indicated that *nad5* (0.06), *nad7* (0.05) and *rps12* (0.05) genes are evolving slowly compared to *nad3* (0.30) and *rps4* (0.23) genes (Supplementary Figure S1).

**Figure 3:**
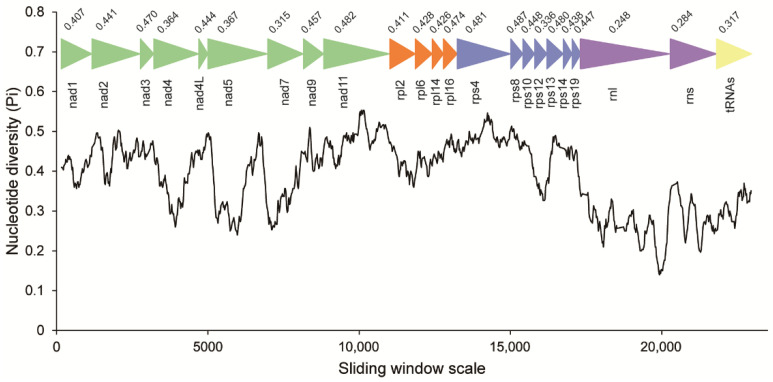
Sliding window analysis of nucleotide diversity between the MRO genes of *Cepedea longa* and *Proteromonas lacertae*. The black curved line represents the value of nucleotide diversity. Gene names, boundaries and average nucleotide diversity values are shown above the line.

Codon usage bias was mostly identical among *C. longa*, *Blastocystis* and *P. lacertae*, except for the most frequent codon for amino acids of Proline and Arginine. The codon TGA for tryptophan and codon CGG/AGG for arginine are unique in *C. longa* among the three Opalinata species. The effective number of codons (Nc) indicates that the usage of synonymous codons in *C. longa* is more balanced than in the other two species (Supplementary Table S4). Concatenated alignments of 20 PCGs, 2 rRNAs and 15 tRNAs of *C. longa* and *P. lacertae* were used to conduct the sliding window analysis. *rnl*, *rns*, *nad4*, *nad5*, *nad7* and *rps12* genes exhibited relatively low sequence variability with Pi values of 0.248, 0.284, 0.364, 0.367, 0.315 and 0.336, respectively. *nad3* (0.470), *nad11* (0.482) and some ribosomal proteins showed high sequence variability (Figure 3). In general, the nucleotide diversity of ribosomal proteins was higher than that of *nad* genes.

### 2.3. Phylogenetic Analyses

**Figure 4:**
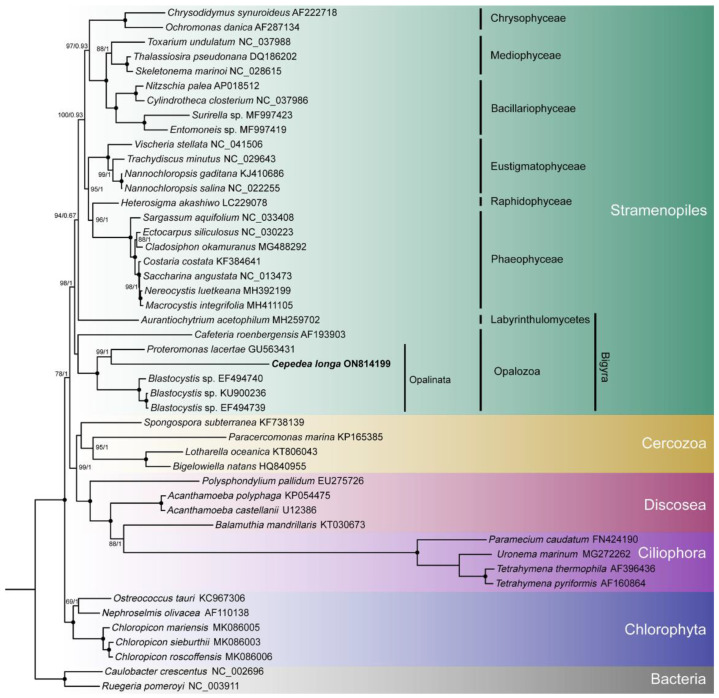
Phylogenetic tree inferred using eight concatenated *nad* sequences. Numbers next to nodes are bootstrap values (ML) and posterior probability values (BI). Black dots represent 100% bootstrap support values and 1.00 posterior probability values. The α-proteobacteria species are set as outgroup taxa. GenBank accession numbers of sequences used in the phylogenetic analyses are listed next to the species name.

Branch topologies produced by ML and BI phylogenetic analyses of concatenated 8 *nad* genes were concordant (Figure 4). Opalinata species were divided into two clades: one containing *Blastocystis* (a representative of Blastocystidae) and the other containing *C. longa* (Opalinea) and *P. lacertae* (Proteromonadea). The species of Opalinata were grouped with high bootstrap support values (100 or 98) and Bayesian posterior probabilities (1.0) (Figure 4). However, the Proteromonadea clade was paraphyletic, due to the Karotomorpha species being resolved as close relatives with Opalinea based on 18S rRNA genes of more species (Supplementary Figure S2). We also found that Bigyra might be a paraphyletic (Figure 4).

**Figure 5:**
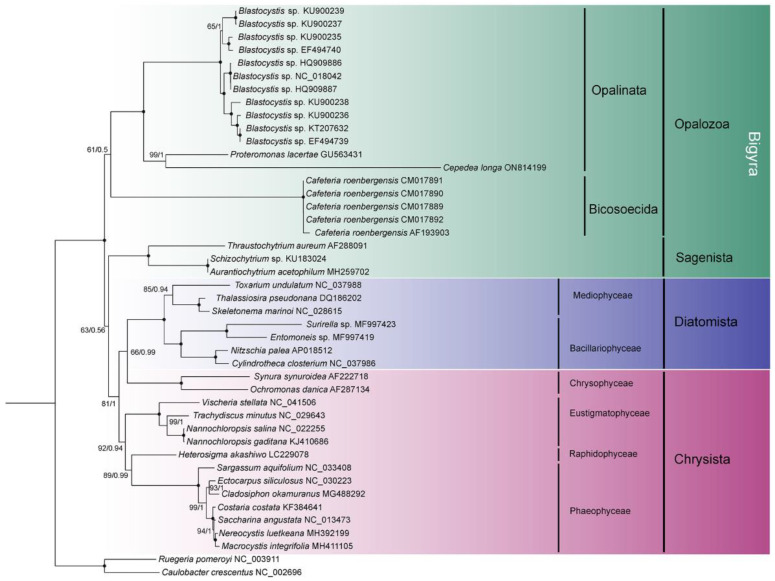
The phylogenetic tree of Stramenopiles inferred using the dataset comprising concatenated *nad* sequences of mitochondrial genomes. Black dots represent 100% bootstrap support values and 1.00 posterior probability values. The tree is rooted using two bacterial species.

To explore the topology of the Bigyra, we focused on the phylogenetic relationships on Stramenopiles species. The results showed that Bigyra was paraphyletic (Figure 5). We also removed fast-evolving amino acid sites from the concatenated sequences and performed phylogenetic analysis anew, but the topology of Bigyra in the ML tree did not change (Supplementary Figure S3). Then we tried to remove fast-evolving taxa (*Cafeteria*) from the dataset, and this resolved monophyletic Bigyra but with a weak support bootstrap value (Supplementary Figure S4).

## 3. Discussion

Mitochondria are known as “powerhouses” that supply cells with energy [1]. Although their types and functions vary substantially among different eukaryotic lineages [5], they are all derived from endosymbiotic α-proteobacteria within an archaeal host cell closely related to the Asgard archaea. However, mitochondrial genomes are vastly reduced in gene content compared to the genomes of α-proteobacterial relatives [40,41]. Stramenopiles, together with Alveolata and Rhizaria, constitute species-rich clades of the super-group SAR [42,43]. The stramenopiles comprise photosynthetic and nonphotosynthetic lineages. All species in Opalinata live as parasites or commensals in the intestinal tracts of amphibians, lizards, birds and mammals [20,27,34]. Although the ultrastructure of the *C. longa* MRO is similar to the standard aerobic mitochondria (double-membrane structure and cristae formed by the inner membrane), it lacks genes encoding the complex III-IV of the electron transport chain (ETC) and ATP synthase; this may be because *C. longa* mainly inhabits the recta with low oxygen concentration [26]. Besides, genes encoding the succinate dehydrogenase (complex II in ETC) were detected in the nuclear genome of *C. longa* (data not shown). This suggests that *C. longa* has a highly reduced ETC, akin to *Blastocystis* [44,45].

Mitochondrial genomes vary extensively in size, structure, organization and gene content among eukaryotes [25]. The IGRs of the MRO genome of *C. longa* are longer than other Opalinata species. Also, it exhibits an inverted repeats structure that was found in another stramenopile, *P. lacertae*. This may have been produced by a recombination and gene inversion event. Similar structures have been observed in other lineages, such as nematodes [46], fishes [47] and birds [48]. We speculate that the IGR between tRNA^Asp^ and *rps13* genes is the most likely origin of replication, as there are five tandem repeat units with 100 bp each in this region, which we called central tandem repeats, and the direction of gene transcription and GC skew values switch from this region. The central tandem repeats were also found in ciliates with linear mitochondrial genomes (e.g., species in Spirotricha), and they are probably associated with replication and transcription initiation [49,50]. We speculate that the central tandem repeats of *C. longa* might play an important role in the direction of gene transcription.

Generally, sequences can evolve under negative selection (Ka/Ks < 1), neutral selection (Ka/Ks = 1) or positive selection (Ka/Ks > 1) [51,52]. Ka/Ks values of PCGs in the *C. longa* MRO genome were all smaller than 1, compared to corresponding genes in Opalinata. This indicates that these PCGs are evolving under negative selection, which is the most prevalent form of selection maintaining the long-term stability of biological structures as it constantly sweeps away deleterious mutations [53]. Biased gene conversion is a recombination-associated evolutionary process that may drive gene evolution, and it tends to increase the G + C content over evolutionary time [54,55]. The G + C content of *C. longa* is higher than that of *P. lacertae* and *Blastocystis*, especially in the G + C content of IGRs (2–3 times as high as in other Opalinata species), which is generally regarded as neutrally evolving positions [52,56].

Pseudogenes are generally defined as nonfunctional sequences originally derived from functional genes [57]. In the *C. longa* MRO genome, two pseudo-*nad3* genes are probably non-functional since the TAG stop codon is located inside the gene, while two pseudo-*nad5* genes possess highly degraded 3′ regions. All pseudogenes have conserved 5′ regions exhibiting high identities with the functional *nad5* and *nad3* genes. We also found the order of genes corresponded to *P. lacerate*; for example, *rps14*-*rps8*-*rpl6* and L-H-C-A-R-*rns*-V. However, the evolutionary routes of gene order rearrangements in Opalinata are still unclear, since the MRO genome data remain scarce or even unavailable for many lineages. To further research this topic, more MRO genomes of Opalinata lineages, such as *Karotomorpha*, *Protoopalina* or *Opalina*, remain to be sequenced, analyzed and compared.

*Blastocystis*, *P. lacerate* and *C. longa* inhabit the intestinal tracts of homeothermic animals, terrestrial ectothermic animals and amphibious ectothermic animals, which represent three different habitats of Opalinata. Although their morphological characteristics and lifestyles are distinctly different, phylogenetic analyses based on eight concatenated *nad* genes indicate that *Cepedea*, *Proteromonas* and *Blastocystis* are phylogenetically closely related. More specifically, *P. lacertae* and *C. longa* were resolved as sister clade with high support based on the MRO *nad* genes. All available 18S rRNA gene sequences from Opalinea and Proteromonadea in the GenBank database were also downloaded to reconstruct the phylogenetic relationships among Opalinata. The results showed that Proteromonadea is paraphyletic: *Proteromonas* was monophyletic, while *Karotomorpha* was closely related to opalinids [32,58]. These relations were also postulated by Patterson [59] via ultrastructural studies on ribbons of microtubules and flagellar transitional regions [24]. Besides, our results also support the monophyly of Opalinea and Blastocystidae.

Phylogenetic analysis of concatenated *nad* genes extracted from mitochondrial genome indicated the paraphyly of Bigyra, comprising Opalozoa and Sagenista, in our study. The topology was in accordance with that of Noguchi et al. [60], Derelle et al. [15] and Cho et al. [61]. Previously, the monophyly of Bigyra was recovered using the dataset of 339 protein alignments when divergent opalozoan lineages (*Blastocystis* and *Cafeteria*) were successively removed, which indicates that long-branch attraction might hamper phylogenetic reconstruction in the Bigyra lineage due to these fast-evolving taxa [15]. Following this evidence, we also attempted to remove *Blastocystis* or *Cafeteria* species in phylogenetic analysis and found that the monophyly of Bigyra lineage was recovered when *Cafeteria* species were removed (Supplementary Figure S4). The topology of the ML tree was congruent with phylogenetic analysis based on a 120-gene dataset [62] and the trees obtained after the removal of divergent taxa [15]. However, it was paraphyletic when *Blastocystis* was removed (Supplementary Figure S5). Although the fast-evolving amino acid sites in the data matrix were removed (using the threshold of 20% conservation value), the tree topology of Bigyra was still paraphyletic. In conclusion, concatenated mitochondrial genes offer weak support for the paraphyly of Bigyra.

## 4. Materials and Methods

### 4.1. Specimen Collection, Identification and Observation

*Cepedea longa* specimens were collected from the recta of frogs *F. limnocharis* (Figure 1A) captured in the Meishan, Sichuan Province, China (30°04′–30°16′ N, 103°53′–104°30′ E). Animals were handled in accordance with the recommended guidelines for animal experimentation by the Chinese Association for Laboratory Animals Sciences, and animal procedures were approved by the Animal Care and Ethics Committee of Institute of Hydrobiology, Chinese Academy of Sciences (project identification code: IHB/LL/2019013). Briefly, all frogs were transported alive into the laboratory for further examination, they were anaesthetized and dissected as soon as possible. Opalinids were collected into Petri dishes with sterile 0.65% saline solution after examination of the recta. *C. longa* cells were transferred to a fresh sterile 0.65% saline solution to remove other opalinids and frog cell contaminants. For MRO fluorescent staining, cells were stained with the MitoTracker Red CMXRos (Meilun Biotechnology, Dalian, China) using the working concentration of 250 nM for 30 min. Then the staining solution was discarded with a micropipette, and cells were again washed with the 0.65% saline solution; finally, cells were stained with DAPI for 10 min. Living specimens and fluorescent-stained cells (579 nm wavelength excitation for MitoTracker Red signals and 359 nm wavelength excitation for DAPI signals) were photographed with a microscope (ZEISS Axio Imager A2, Carl Zeiss, Jena, Germany) equipped with a digital camera (sCMOS PCO, Kelheim, Germany). For transmission electron microscopy, specimens were fixed in 2.5% glutaraldehyde in 0.2 M phosphate-buffered saline (PBS, pH7.4) at 4°C for 24 h; then post-fixed in 2% osmium tetroxide with PBS at 4°C for 3 h; followed by dehydration in a gradient series of acetone; and finally embedded in Araldite. Ultra-thin sections were cut and stained with uranyl acetate and lead citrate before being observed and photographed using an HT-7700 transmission electron microscope (Hitachi, Tokyo, Japan).

### 4.2. DNA Extraction, MRO Genome Sequencing and Assembly

Cells were preserved in absolute ethanol and stored at −20°C. The total genomic DNA was extracted from preserved *C. longa* cells (~10,000 cells) for the Illumina sequencing and Sanger sequencing using a standard phenol/chloroform method. The next-generation sequencing library was constructed using the NexteraXT DNA Library Preparation Kit (illumina, CA, USA) and sequenced on the Illumina Novaseq platform (San Diego, CA, USA). For the MRO genome assembly, after trimming the adapters and filtering low-quality reads (reads with 5% unidentified nucleotides and with quality values below 20), the resulting clean reads were assembled de novo using software SPAdes v3.14.1 set as the default parameters. The contigs were aligned to MRO genome sequences of *Blastocystis* and *P. lacerate* using blastn v2.9.0 with the e-value < 1 × 10^−5^. The probable MRO genome sequences of *C. longa* were filtered, contigs extended using PRICE (Paired-Read Iterative Contig Extension) [63] and mapped with the clean reads using bowtie2 [64]. Finally, the results were assembled de novo again using SPAdes 3.14.0 [65]. The process was repeated until the total size of the draft MRO genome stabilized. To verify the MRO genome sequence of *C. longa*, “primer walking” and Sanger sequencing were conducted according to the draft MRO genome generated from next-generation sequences assembly.

### 4.3. MRO Genome Annotation and Analysis

The protein-coding genes (PCGs), transfer RNAs (tRNAs) and large/small rRNA subunit genes (*rnl*/*rns*) of the MRO genome of *C. longa* were initially annotated using the MFannot tool (https://megasun.bch.umontreal.ca/apps/mfannot/ (accessed on 24 May 2020)) using the genetic code 4. The tRNA gene prediction was additionally performed using tRNAscan-SE [66] and RNAweasel (https://megasun.bch.umontreal.ca/cgi-bin/RNAweasel/RNAweaselInterface.pl (accessed on 25 May 2020)). The final tRNA prediction results were confirmed if they were predicted by all three tools. The boundaries of protein open reading frames (ORFs) were inferred with the help of NCBI’s ORF Finder, applying the genetic code 4 (https://www.ncbi.nlm.nih.gov/orffinder/ (accessed on 30 May 2020)). PCGs were identified based on NCBI’s BLAST homology searches with ORFs. We also used the HHpred web server and UniProt BLAST (Pfam and UniProtKB/Swiss-Prot were selected as target databases, respectively) to identify and confirm the PCGs [67,68]. PCGs that could not be identified with confidence were annotated as hypothetical proteins. The *rnl* and *rns* genes were verified by aligning them with close relatives *Blastocystis* and *P. lacerate*.

The MRO genome map was created using Circos v0.69-8 [69]. Eight completely sequenced mitochondrial genomes of stramenopiles were chosen to compare gene contents. Codon usage was calculated in the MRO genomes of *C. longa*, *Blastocystis* sp. and *P. lacerate* and compared using the CodonW program version 1.4.2. The KaKs_Calculator was used to estimate selective pressure on PCGs [51]. The sliding window analysis was conducted using DnaSP v6 [70], with a sliding window of 300 bp and a step size of 20 bp implemented to estimate the nucleotide divergence between *C. longa* and *P*. *lacertae*.

### 4.4. Phylogenetic Analyses

Phylogenetic analyses were conducted using the newly sequenced *C. longa* MRO genome and 44 mitochondrial genome sequences from other stramenopiles, as well as some other eukaryotic and prokaryotic organisms, which were retrieved from the Genbank. Two species of prokaryotes, *Caulobacter crescentus* and *Ruegeria pomeroyi*, were set as outgroups. Eight *nad* subunit protein sequences were aligned using the MAFFT v7.313 [71] plugin in PhyloSuite v1.2.1 [72] and then concatenated in the order *nad1*, *nad2*, *nad3*, *nad4*, *nad4L*, *nad5*, *nad7*, *nad9* using the “Concatenate Sequence” function in PhyloSuite. The best partitioning scheme and the most appropriate evolutionary models were selected by PartitionFinder2 [73] with a greedy algorithm and AICc criterion. Phylogenetic analyses were conducted using two programs implemented in PhyloSuite: IQ-Tree v1.6.8 [74] was used for the Maximum likelihood (ML) analysis, and Bayesian inference (BI) analysis was performed using MrBayes v3.2.6 [75]. The ML analysis was conducted with the LG + I + G + F model of amino acid evolution and 5000 ultrafast bootstrap replicates, whereas the BI analysis was performed with 2,000,000 MCMC generations where trees were sampled every 1000 generations and the burn-in set at 25% (500,000 samples). Finally, trees were visualized by Figtree v1.4.0 and further edited in iTOL [76].

We also conducted phylogenetic analyses on Opalinata using 18S rRNA sequences: 32 sequences of Opalinata and 3 sequences of *Cafeteria* were obtained from GenBank (among these, the 18S rRNA sequence of *C. longa* was submitted by us in our previous study). The phylogenetic trees were reconstructed using ML and BI methods.

To verify the topology of the Bigyra lineage, a total of 44 stramenopiles affiliated to the Bigyra (Opalozoa + Sagenista) and Gyrista (Diatomista + Chrysista), with a bacterial outgroup, were selected to reconstruct the phylogenetic tree using the ML method in IQ-Tree. Bayesian inference was performed using the site-heterogeneous mixture model in PhyloBayes-mpi version 1.8 [77]. Two independent runs were performed until the two chains converged (a threshold of maxdiff < 0.1). Fast-evolving sites were removed using the trimAl v1.2 [78] by setting the threshold to the conservation value of 80%.

## 5. Conclusions

In this study, the opalinid *C. longa* inhabiting the recta of *F. limnocharis* were collected to sequence their MRO genome, which is the first MRO genome reported within the opalinid lineage. Its MRO genome was the longest in Opalinata (to date), with two inverted repeat structures. Gene rearrangements seem to exist within the Opalinata, but this needs to be further clarified by sequencing further MRO genomes in this lineage. Although three Opalinata species have different morphological characteristics, phylogenetic analyses based on eight concatenated *nad* genes indicate that they are close relatives. Phylogenetic analyses based on the 18S rRNA gene also support the monophyly of Opalinea and Blastocystidae and the paraphyly of Proteromonadea. Besides, our dataset and analyses offered weak support for the paraphyly of Bigyra.

## Figures and Tables

**Table 1:** Comparison of characteristics of MRO genomes of *Cepedea longa* and other Opalinata species.

	*Cepedea longa*	*Proteromonas lacertae*	*Blastocystis* sp. NandII	*Blastocystis* sp. Strain Flemming	*Blastocystis* sp. Strain F5323
Total length (bp)	62,395	48,663	28,385	28,305	28,788
Total ORFs length (bp)	38,313	34,569	21,966	21,594	21,909
Total intergenic regions length (bp)	11,510	2340	1162	1046	1225
G + C content (%)					
Total	25.7	22.7	19.9	19.7	18.9
Intergenic regions (IGRs)	25.4	12.0	10.8	11.9	8.4
Protein-coding regions	23.3	19.0	17.9	17.8	17.1
Structural RNA genes	34.6	35.4	30.0	29.4	29.0
Gene number					
Protein coding	40	39	27	26	26
Structural RNAs	45	49	18	18	17
Average ORFs length (bp)	958	886	813	830	843
Average IGRs length (bp)	151	31	33	29	40

## Data Availability

The raw paired-end reads in this study were deposited in the NCBI SRA with BioProject ID: PRJNA850552. MRO genome sequence of *Cepedea longa* was deposited in Genbank under the accession number ON814199.

## Supplementary Materials

The following supporting information can be downloaded at: https://www.mdpi.com/article/10.3390/ijms232113472/s1.
