# Supplementary material for: The Genome of the Mitochondrion-Related Organelle in Cepedea longa, a Large Endosymbiotic Opalinid Inhabiting the Recta of Frogs
Source: Int J Mol Sci. 2022 Nov 3;23(21):13472. doi: 10.3390/ijms232113472 (PMC9656049; doi:10.3390/ijms232113472)
Supplement: Supplementary file 1 [file ijms-23-13472-s001.zip › ijms-1927178-supplementary.pdf]

Supplementary Materials

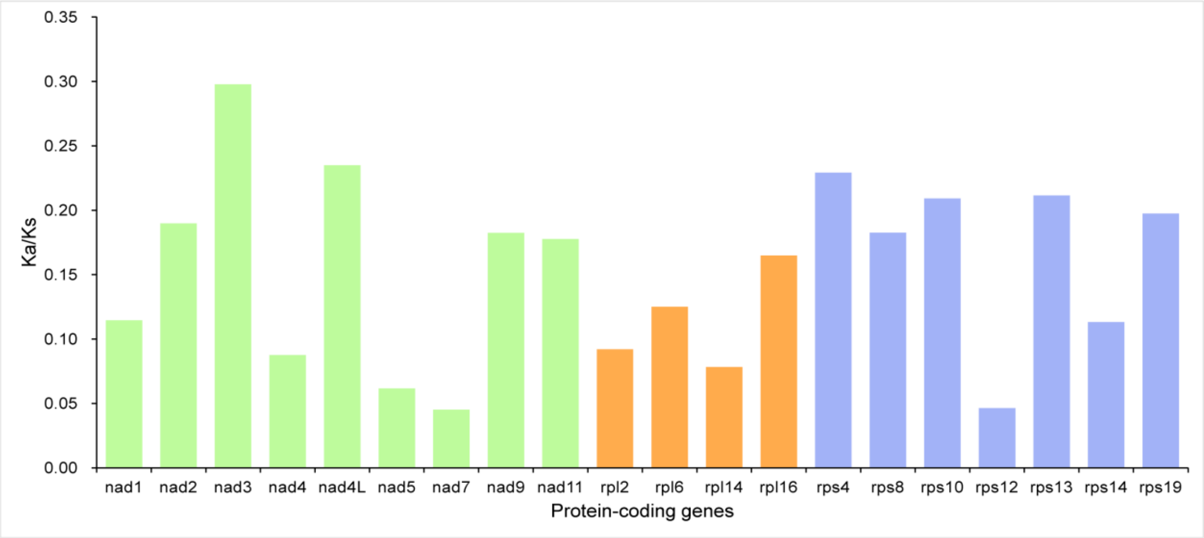

Figure S1. The ratios of non-synonymous to synonymous substitution rates for protein-coding genes of *Cepedea longa* and *Proteromonas lacertae*.

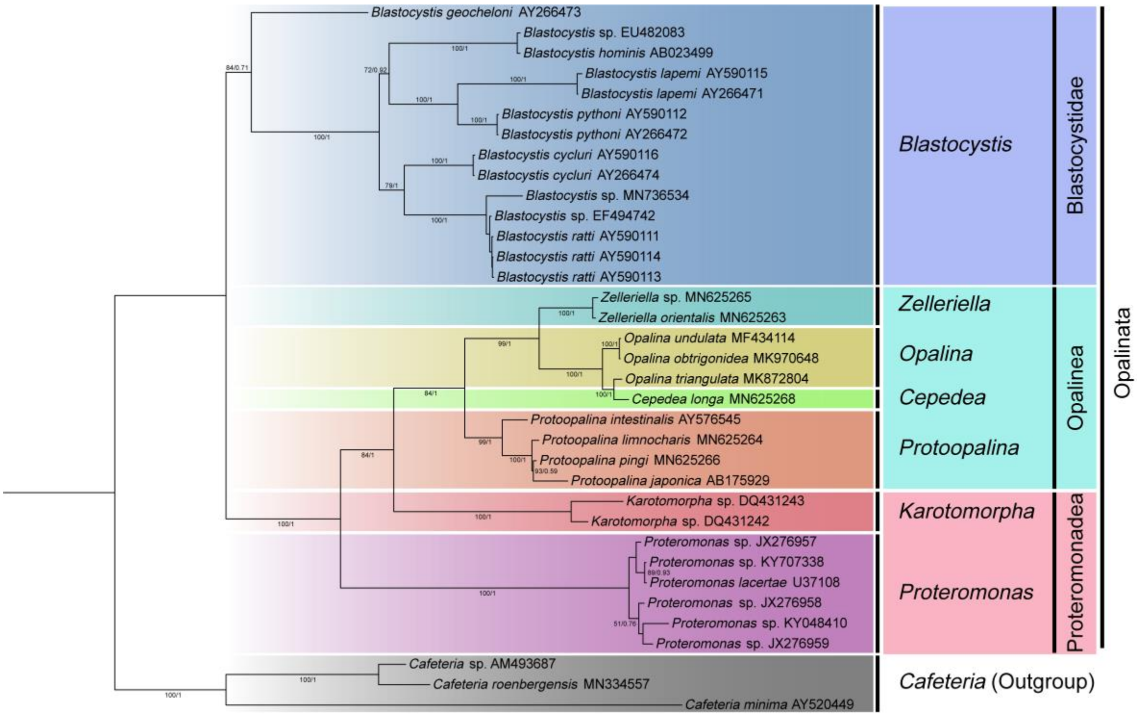

Figure S2. Phylogenetic relationships in the Opalinata lineage inferred using the 18S ribosomal gene. Numbers beside the nodes are bootstrap values (ML) and posterior probability values (BI). Genbank accession numbers of sequences used in the phylogenetic analyses are listed next to the species names.

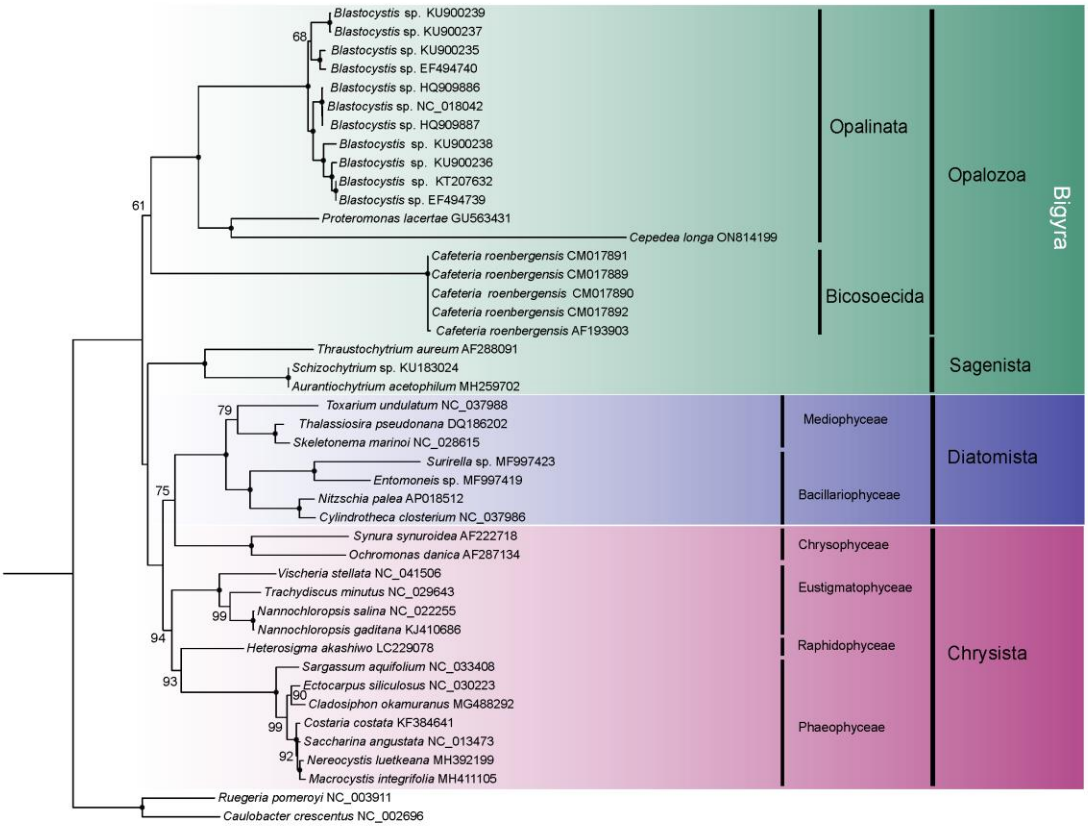

Figure S3. The Maximum-Likelihood phylogenetic tree of Stramenopiles inferred from conserved sites of concatenated *nad* sequences of the mitochondrial genome. Black dots represent 100% bootstrap support values. The tree is rooted using two bacteria species.

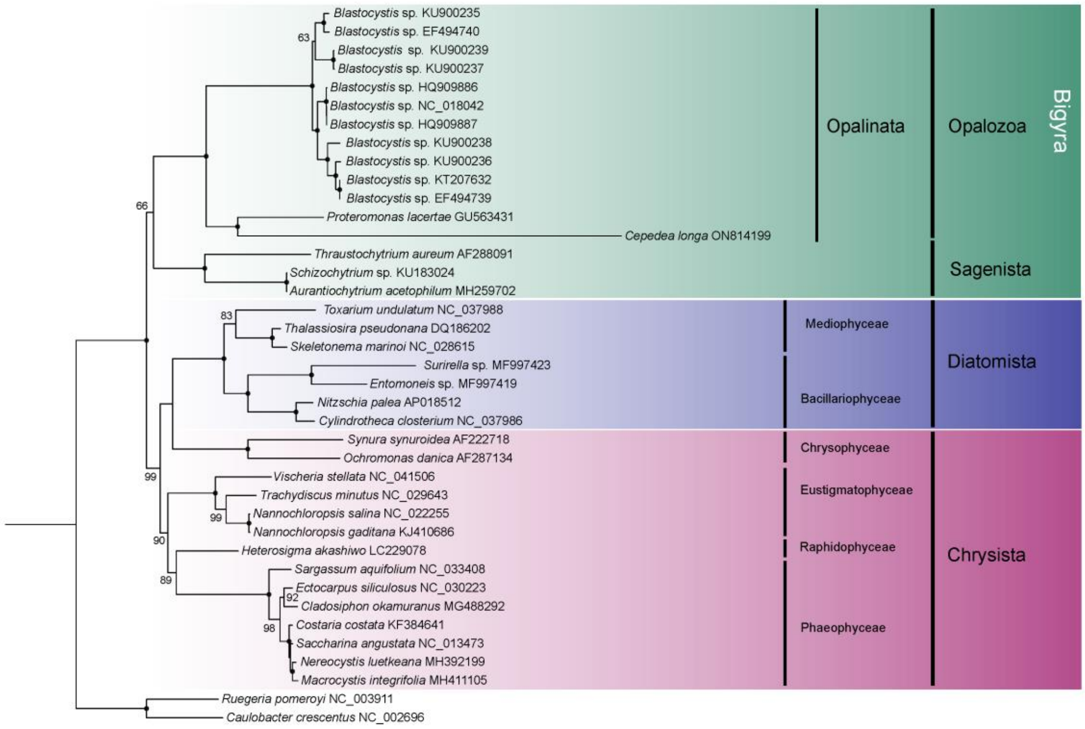

Figure S4. The phylogenetic tree of Stramenopiles inferred using the dataset comprised of concatenated *nad* sequences of mitochondrial genomes, with *Cafeteria* species pruned from the dataset. Black dots represent 100% bootstrap support values and 1.00 posterior probability values. The tree is rooted using two bacteria species.

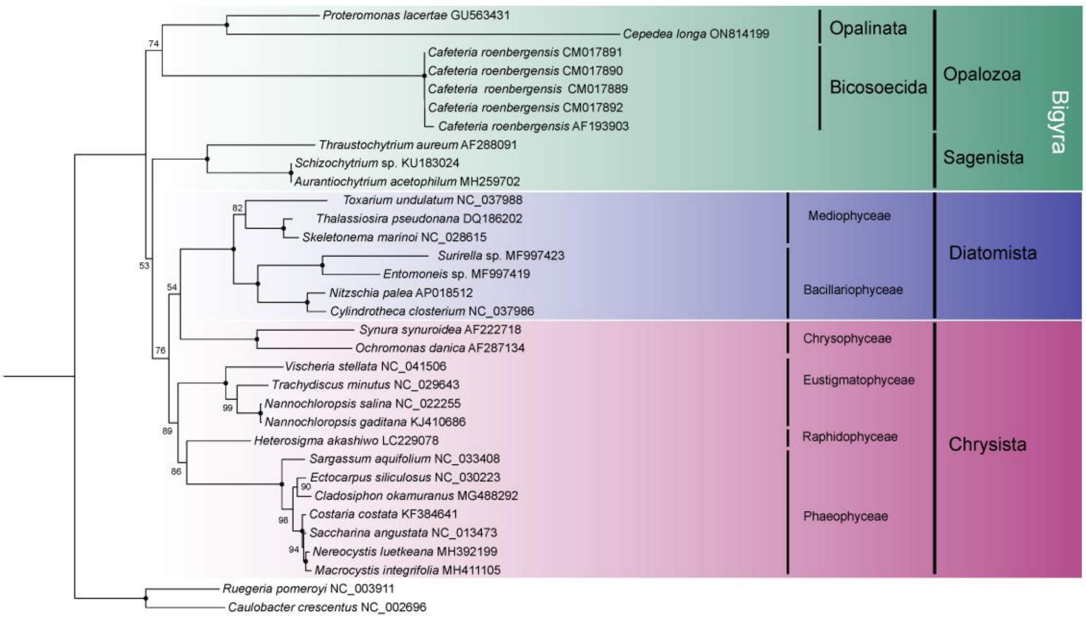

Figure S5. The Maximum-likelihood phylogenetic tree of Stramenopiles inferred using a dataset comprised of concatenated *nad* sequences of the mitochondrial genome, but with *Blastocystis* species pruned from the dataset. Black dots represent 100% bootstrap support values. The tree is rooted using two bacteria species.

Table S1. The annotated MRO genome of *Cepedea longa*

Gene	Position		Direction	GC content (%)	Length		Codon	
	From	To			nt	aa	Start	Stop
I	49	121	-	31.51	73			
L	138	220	-	33.73	83			
nad2	228	1,793	-	22.03	1,566	522	ATG	TAG
K	1,795	1,866	-	38.89	72			
M	1,878	1,949	-	33.33	72			
K	2,016	2,086	-	39.44	71			
nad4L	2,094	2,381	-	20.83	288	96	ATG	TAG
Y	2,382	2,463	-	35.37	82			
rnl	2,501	5,264	-	33.36	2,764			
V	5,433	5,505	-	34.25	73			
rns	5,506	6,851	-	36.48	1,346			
R	6,885	6,957	-	32.88	73			
A	6,981	7,051	-	28.17	71			
C	7,052	7,122	-	30.99	71			
H	7,124	7,196	-	34.25	73			
L	7,343	7,415	-	35.62	73			
M	7,417	7,487	-	32.39	71			
L	8,128	8,209	-	39.02	82			
G	8,217	8,287	-	43.66	71			
F	8,317	8,388	-	43.06	72			
nad5	8,398	10,404	-	24.61	2,007	669	ATG	TAA
pseudo-nad5	10,870	11,312	-	19.86	443		ATG	
nad4	11,595	13,076	-	23.08	1,482	494	ATG	TAA
nad3	13,378	13,803	-	22.07	426	142	ATG	TAA
pseudo-nad3	13,936	14,268	-	18.92	333		ATG	
orf254	14,201	14,965	-	20.00	765	255	ATG	TAG
orf209	15,117	15,746	-	19.52	630	210	ATG	TAA
Q	16,193	16,264	-	40.28	72			
W	16,641	16,711	-	26.76	71			
nad7	17,146	18,336	-	31.57	1,191	397	ATG	TAA
orf234	18,371	18,772	-	27.11	402	134	ATG	TAA
nad1	18,923	19,894	-	26.03	972	324	ATG	TAA
rps12	19,923	20,333	-	28.71	411	137	ATG	TAA
E	20,336	20,407	-	36.11	72			
P	20,437	20,509	-	31.51	73			
orf388	20,535	21,701	-	22.28	1,167	389	ATG	TAA
orf185	21,698	22,246	-	20.61	558	186	ATG	TAA
rpl14	22,249	22,629	-	28.61	381	127	ATG	TAA
rpl2	22,636	23,403	-	29.30	768	256	ATG	TAG
rps4	23,408	27,535	-	22.19	4,128	1376	ATG	TAA

Table S1 continued

Gene	Position		Direction	GC content (%)	Length		Codon	
	From	To			nt	aa	Start	End
orf233	27,553	28,473	-	18.35	921	307	TTG	TAG
rpl6	28,533	29,075	-	20.21	564	188	ATG	TAG
rps8	29,075	29,473	-	22.81	399	133	ATG	TAA
rps14	29,483	29,782	-	26.33	300	100	ATG	TAG
nad11	29,843	31,870	-	22.73	2,028	676	ATG	TAA
rps11	31,872	33,779	-	20.96	1,908	636	ATG	TAG
rps13	33,751	34,284	-	20.79	534	178	ATG	TAA
D	34,955	35,026	+	31.94	72			
rps19	35,027	35,311	+	26.67	285	95	ATG	TAA
orf291	35,312	36,187	+	17.24	876	292	ATG	TAA
orf588	36,454	38,220	+	20.09	1,767	589	ATG	TAA
rpl16	38,228	38,674	+	29.53	447	149	GTG	TAA
nad9	39,822	40,472	+	28.11	651	217	ATG	TAA
rps10	40,482	40,862	+	19.95	381	127	ATG	TAA
P	40,821	40,893	+	31.51	73			
E	40,923	40,994	+	37.50	72			
rps12	40,997	41,407	+	29.44	411	137	ATG	TAG
nad1	41,436	42,407	+	25.41	972	324	ATG	TAA
orf234	42,558	42,959	+	26.87	402	134	ATG	TAA
nad7	43,428	44,618	+	31.91	1,191	397	ATG	TAA
W	45,054	45,124	+	25.35	71			
Q	45,500	45,571	+	40.28	72			
orf209	46,317	46,946	+	19.37	630	210	ATG	TAA
orf254	47,098	47,862	+	20.00	765	255	ATG	TAG
pseudo-nad3	47,795	48,127	+	18.62	333		ATG	
nad3	48,260	48,685	+	21.83	426	142	ATG	TAA
nad4	48,987	50,468	+	23.68	1,482	494	ATG	TAG
pseudo-nad5	50,752	51,194	+	19.64	443		ATG	
nad5	51,661	53,667	+	24.61	2,007	669	ATG	TAA
F	53,677	53,748	+	43.06	72			
G	53,778	53,848	+	43.66	71			
L	53,856	53,937	+	39.02	82			
M	54,949	55,019	+	32.39	71			
L	55,021	55,093	+	35.62	73			
H	55,240	55,312	+	34.25	73			
C	55,314	55,384	+	30.99	71			
A	55,385	55,455	+	28.17	71			
R	55,479	55,551	+	32.88	73			
rns	55,585	56,930	+	36.55	1,346			
V	56,931	57,003	+	34.25	73			

Table S1 continued

Gene	Position		Direction	GC content (%)	Length		Codon	
	From	To			nt	aa	Start	End
rnl	57,172	59,933	+	33.42	2,762			
Y	59,971	60,052	+	35.37	82			
nad4L	60,053	60,340	+	20.49	288	96	ATG	TAG
K	60,348	60,418	+	39.44	71			
M	60,485	60,556	+	33.33	72			
K	60,568	60,639	+	37.50	72			
nad2	60,641	62,206	+	21.84	1,566	522	ATG	TAG
L	62,214	62,296	+	33.73	83			
I	62,313	62,385	+	31.51	73			

Table S2. Comparative gene content in eight sequenced mitochondria genomes of stramenopiles[a]

Gene	*Cafeteria roenbergensis*	*Heterosigma akashiwo*	*Chloropicon mariensis*	*Thalassiosira pseudonana*	*Ochromonas danica*	*Blastocystis* sp.	*Proteromonas lacertae*	*Cepedea longa*
rns, rnl	●	●	●	●	●	●	●	●
trnA-trnY	22	26	25	25	24(29)	15(16)	23(45)	17(41)
cob	●	●	●	●	●	○	○	○
cox1-cox3	●	●	●	●	●	○	○	○
nad1	●	●	●	●	●	●	●	●
nad2	●	●	●	●	●	●	●	●
nad3	●	●	●	●	●	●	●	●
nad4	●	●	●	●	●	●	●	●
nad4L	●	●	●	●	●	●	●	●
nad5	●	●	●	●	●	●	●	●
nad6	●	●	●	●	●	●	●	○
nad7	●	●	●	●	●	●	●	●
nad9	●	●	●	●	●	●	●	●
nad10	○	○	●	○	○	○	○	○
nad11	●	●	○	●	●	●	●	●
atp1	●	○	●	○	○	○	○	○
atp4	○	○	●	○	○	○	○	○
atp6	●	●	●	●	●	○	○	○
atp8	●	●	●	●	●	○	○	○
atp9	●	●	●	●	●	○	○	○
rps2	●	●	●	●	●	○	●	○
rps3	●	●	●	●	●	●	○	○
rps4	●	●	●	●	●	●	●	●

Table S2 continued

Gene	*Cafeteria roenbergensis*	*Heterosigma akashiwo*	*Chloropicon mariensis*	*Thalassiosira pseudonana*	*Ochromonas danica*	*Blastocystis* sp.	*Proteromonas lacertae*	*Cepedea longa*
rps7	○	●	●	●	●	○	○	○
rps8	●	●	●	●	●	●	●	●
rps10	○	●	●	●	●	●	●	●
rps11	○	●	●	●	●	●	○	●
rps12	●	●	●	●	●	●	●	●
rps13	●	●	●	●	●	●	●	●
rps14	●	●	●	●	●	●	●	●
rps19	●	●	●	●	●	●	●	●
rpl2	●	●	○	●	●	●	●	●
rpl5	○	●	●	●	○	○	●	○
rpl6	●	●	○	●	●	●	●	●
rpl14	●	●	●	●	●	●	●	●
rpl16	●	●	●	●	●	●	●	●
ORFs	4	4	0	1	9(11)	4	4(5)	8(11)

Filled circle = gene present; open circle = genes absent. Figures in parentheses represent the total gene number, including gene copies.

[a] Genbank accession numbers of 8 species: *Cafeteria roenbergensis*, AF193903; *Heterosigma akashiwo*, LC229078; *Chloropicon mariensis*, MK086005; *Thalassiosira pseudonana*, DQ186202; *Ochromonas danica*, AF287134; *Blastocystis* sp., EF494740; *Proteromonas lacerate*, GU563431; *Cepedea longa*, ON814199

Table S3. Repeats in intergenic regions of the MRO genome of *Cepedea longa*

repeat region	repeat unit size (bp)	unit number	A+T content (%)	corresponding IGR length
1	188	4	61.17	1011
2	13	2	76.92	151
3	23	2	90.91	150
4	100	5	65	670
5	23	3	100	150
6	13	2	76.92	151
7	188	2	61.17	640

Table S4. Comparative codon usage in the MRO PCGs of *Cepedea longa*, *Blastocystis* sp. and *Proteromonas lacertae*

Amino acid	Codon	*C. longa*		*Blastocystis* sp.		*P. lacertae*	
		count	ratio	count	ratio	count	ratio
Phe	UUU	2028	0.97	487	0.86	1005	0.94
	UUC	61	0.03	77	0.14	60	0.06
Leu	UUA	1180	0.62	808	0.90	1205	0.84
	UUG	512	0.27	21	0.02	21	0.01
	CUU	126	0.07	63	0.07	149	0.10
	CUC	3	0.00	4	0.00	0	0.00
	CUA	50	0.03	2	0.00	61	0.04
	CUG	29	0.02	0	0.00	3	0.00
Ile	AUU	853	0.65	559	0.63	1040	0.75
	AUC	45	0.03	39	0.04	28	0.02
	AUA	416	0.32	287	0.32	322	0.23
Cys	UGU	355	0.90	82	0.87	166	0.91
	UGC	39	0.10	12	0.13	17	0.09
Val	GUU	539	0.62	174	0.54	383	0.70
	GUC	23	0.03	13	0.04	4	0.01
	GUA	229	0.26	134	0.41	146	0.27
	GUG	78	0.09	3	0.01	14	0.03
Ser	UCU	292	0.34	211	0.49	372	0.46
	UCC	28	0.03	9	0.02	16	0.02
	UCA	136	0.16	73	0.17	202	0.25
	UCG	107	0.12	1	0.00	6	0.01
	AGU	277	0.32	131	0.30	200	0.25
	AGC	30	0.03	6	0.01	16	0.02
Pro	CCU	86	0.44	60	0.43	135	0.57
	CCC	8	0.04	0	0.00	12	0.05
	CCA	75	0.38	80	0.57	85	0.36
	CCG	27	0.14	1	0.01	4	0.02
Thr	ACU	144	0.46	189	0.55	286	0.61
	ACC	18	0.06	14	0.04	11	0.02
	ACA	100	0.32	139	0.41	168	0.36
	ACG	50	0.16	0	0.00	7	0.01

Table S4 continued

Amino acid	Codon	*C. longa*		*Blastocystis* sp.		*P. lacertae*	
		count	ratio	count	ratio	count	ratio
Glu	GAA	220	0.71	170	1.00	256	0.94
	GAG	91	0.29	0	0.00	17	0.06
Ala	GCU	181	0.55	131	0.58	283	0.74
	GCC	10	0.03	3	0.01	9	0.02
	GCA	104	0.32	88	0.39	89	0.23
	GCG	32	0.10	2	0.01	4	0.01
Tyr	UAU	916	0.96	570	0.96	685	0.96
	UAC	36	0.04	26	0.04	26	0.04
His	CAU	182	0.96	78	0.93	171	0.90
	CAC	8	0.04	6	0.07	19	0.10
Gln	CAA	136	0.69	163	1.00	234	0.97
	CAG	60	0.31	0	0.00	7	0.03
Asn	AAU	665	0.95	779	0.94	1163	0.97
	AAC	38	0.05	46	0.06	34	0.03
Lys	AAA	583	0.78	717	0.99	1012	0.98
	AAG	167	0.22	5	0.01	17	0.02
Asp	GAU	345	0.95	195	0.99	286	0.96
	GAC	20	0.05	1	0.01	11	0.04
Met	AUG	316	1.00	130	1.00	181	1.00
Trp	UGA	6	0.03	0	0.00	0	0.00
	UGG	170	0.97	42	1.00	69	1.00
Arg	CGU	258	0.43	87	0.43	145	0.47
	CGC	29	0.05	1	0.00	8	0.03
	CGA	41	0.07	11	0.05	10	0.03
	CGG	35	0.06	0	0.00	0	0.00
	AGA	161	0.27	103	0.51	148	0.48
	AGG	80	0.13	0	0.00	0	0.00

Table S4 continued

Amino acid	Codon	*C. longa*		*Blastocystis* sp.		*P. lacertae*	
		count	ratio	count	ratio	count	ratio
Gly	GGU	345	0.58	256	0.98	276	0.61
	GGC	17	0.03	4	0.02	8	0.02
	GGA	95	0.16	2	0.01	154	0.34
	GGG	138	0.23	0	0.00	18	0.04
Nc[a]		36.74		29.12		29.28	

[a] The effective number of codons
